# The patient experience of fatigue in motor neurone disease

**DOI:** 10.3389/fpsyg.2013.00788

**Published:** 2013-10-25

**Authors:** Chris J. Gibbons, Everard W. Thornton, Carolyn A. Young

**Affiliations:** ^1^NIHR Collaboration for Leadership in Applied Health Research and Care, Centre for Primary Care, The University of ManchesterManchester, UK; ^2^The Walton Centre for Neurology and NeurosurgeryLiverpool, UK; ^3^Department of Experimental Psychology, Institute of Psychology, Health and Society, University of LiverpoolLiverpool, UK

**Keywords:** fatigue, motor neurone disease (MND), amyotrophic lateral sclerosis (ALS), qualitative

## Abstract

**Aims:** This paper is a qualitative investigation that aims to investigate the lived experience of fatigue in patients with motor neurone disease—a progressive and fatal neurological condition.

**Background:** Fatigue is a disabling symptom in motor neurone disease (MND) that affects a large number of patients. However, the term “fatigue” is in itself imprecise, as it remains a phenomenon without a widely accepted medical definition. This study sought to investigate the phenomenon of fatigue from the perspective of the MND patient.

**Methods:** Ten patients with MND participated in semi-structured recorded interviews at a regional neuroscience center in Liverpool, UK. Transcripts analysis was broadly informed by the principles of interpretative phenomenological analysis (IPA).

**Findings:** Fatigue was unanimously explained to be disabling and progressive phenomenon. Participants described two forms of fatigue: whole-body tiredness or use-dependent reversible muscle weakness related to exertion of limb and bulbar muscles. Both weakness and whole-body tiredness could be experienced simultaneously, and patients used the terms “fatigue” and “tiredness” interchangeably. Alongside descriptions of fatigue themes of Adaptation, Motivation, Avoidance, Frustration and Stress were revealed. Fatigue could be defined as “reversible motor weakness and whole-body tiredness that was predominantly brought on by muscular exertion and was partially relieved by rest.”

**Conclusion:** The results of this study support a multi-dimensional model of fatigue for patients with MND. Fatigue appears to be experienced and explained in two ways, both as an inability to sustain motor function and as a pervasive tiredness. Fatigue was only partially relieved by rest and tended to worsen throughout the day. It is crucial that MND care practitioners and researchers appreciate the semantic dichotomy within fatigue.

## Introduction

MND is a progressive and fatal neurodegenerative disease without a known cure or effective treatment. It is clinically characterized by progressive muscular weakness leading to death by respiratory insufficiency, usually within 3 years of symptom onset (Haverkamp et al., 1995). Motor neurone disease usually manifests itself as muscular wasting and weakness in limb or bulbar regions (Talbot, 2002). Incidence of the disease is about 50% higher in men than in women, with rates of 2.6 per 100,000 for women and 3.9 per 100,000 for men in the UK (Alonso et al., 2009).

## Background

Fatigue is one of the most commonly reported symptoms in MND (Lou et al., 2003; Ramirez et al., 2008), with one study reporting prevalence of clinically significant fatigue to be present in 44% of patients (McElhiney et al., 2009). The aetiology of fatigue is not fully understood and symptom salience varies between individuals. Fatigue has been associated with depression, reduced quality of life and poor functional status (Lou et al., 2003; McElhiney et al., 2009; Gibbons et al., 2013), though there is some debate as to its precise relationship with concomitant disease factors, including depression (Ramirez et al., 2008; Gibbons et al., 2011a).

The experience of fatigue is ubiquitous and a fundamental part of the human condition. In medicine, fatigue is one of the most commonly reported symptoms by patients, with a prevalence up to 45% in primary care (Lewis and Wessely, 1992). The term “fatigue” is commonly used, though it is not well-defined as the distinct experiences of tiredness, exhaustion, weakness, and weariness may all be described as “fatigue.”

Ream and Richardson (Ream and Richardson, 1996) provided a definition of fatigue for nursing use, suggesting that it was “a subjective, unpleasant symptom which incorporates total body feelings ranging from tiredness to exhaustion creating an unrelenting overall condition which interferes with individuals' ability to function to their normal capacity.” (p. 527).

This definition is useful, however, its relevance to patients with MND, whose functional capacity may be severely limited and for whom “exhaustion” may mean more than simply “extreme tiredness,” is unclear. The experience of fatigue may differ significantly between different diagnoses and within different neurological conditions. In multiple sclerosis fatigue is characterized as extreme feelings of constant tiredness, including cognitive, motor and motivational elements (Mills and Young, 2008). However, in neurological diseases of the peripheral nervous system, such as myasthenia gravis; fatigue is understood purely to be a progressive loss of strength, known as fatigability (Chaudhuri and Behan, 2004).

As fatigue may present in different ways, and can vary even within neurological disorders, it is important to develop a greater understanding of how the concept of MND fatigue is understood from the perspective of the patients that experience it firsthand. Determining patients' conceptualization of fatigue is a foundation stone for developing a greater understanding of this subjective symptom, and a gateway to developing successful treatments and carrying out more useful and valid research.

## The study

### Aim

The impetus of the current study is to provide both a phenomenological account of fatigue in motor neurone disease and offer a definition that may be used as the basis for future qualitative work to develop a disease-specific questionnaire for fatigue.

### Methodology

An ideal tool to explore the subjective experience of fatigue is Interpretative Phenomenological Analysis (IPA) (Smith and Osborn, 2003), a structured qualitative approach that is suitable for discovering how individuals perceive the “*particular situations they are facing, how they are making sense of their personal and social world. IPA is especially useful when* one *is concerned with complexity, process, or novelty*” (p. 55). Qualitative methodologies have been used to expand knowledge of fatigue from a patient perspective in other neurological disorders (Mills and Young, 2008), and cancer (Magnusson et al., 1999) and IPA has emerged as a methodology that allows for an in-depth exploration of meaning and experience for individuals experiencing fatigue (Jorgensen, 2006).

### Participants

In accordance with IPA guidelines (Smith and Osborn, 2003) purposive sampling was employed in this study. This method ensures that “information rich” cases are examined which can offer the greatest insight into the research question (Devers and Frankel, 2000). As such, participants were deemed suitable for inclusion to the study if they had explained that they felt fatigued as part of a holistic assessment of symptoms carried out by a consultant neurologist. A sample size of 10 patients was decided to be sufficient to explore the symptom of fatigue in this population. Ten fatigued patients were approached that were deemed to have sufficient variation in age, gender and disease experience to maximize the variation in the context that fatigue may occur within this population.

No additional participants were interviewed as the experience of fatigue was exhaustively described, with apparent saturation of themes in the final interviews. Ethical approval was provided by Tayside Medical Research Ethics (07/S1402/64) and local research governance committees.

### Data collection

Participants were approached at a regional neuroscience center in Liverpool, UK. During either a routine clinic appointment or during an MND information session run in the center. All participants had expressed fatigue to be a relevant issue prior to inclusion in the study. Participants all had a confirmed diagnosis of MND from a neurologist with a special interest in MND. Interviews were conducted following the clinic appointment or arrangements were made for an interview to be conducted at the participant's home. Spouses or lay carers were allowed to be present if requested but were encouraged to be observers. Patients gave informed consent prior to undertaking the interview.

Demographic and medical details were taken for all patients in the study including scores for anxiety and depression, using the Hospital Anxiety and Depression Scale (HADS) (Zigmond and Snaith, 1983). Functional status scores measured by the Amyotrophic Lateral Sclerosis Functional Rating Scale—Revised (ALSFRS-R) (Cedarbaum et al., 1999) were recorded within 4 weeks of the interview.

### Data collection: interview protocol

Participants took part in a confidential semi-structured interview that lasted between 15 and 45 min. Patients were initially asked to describe fatigue in their own words. The interviews were participant-led but a skeletal structure of open-ended questions was devised to explore areas of fatigue including: functional limitations, cognitive effects, fatigue development, social effects, negative affect, learned responses and relieving factors. The skeletal structure was informed by prior research investigating fatigue for patients with multiple sclerosis (Mills and Young, 2008). Interviews were anonymously recorded on cassette tape and kept in a locked filing cabinet in the study center. Taped interviews were transcribed anonymously and then given a false name of the correct sex. The names used in this report are not the real names of the patients enrolled in the study.

### Data analysis

Interviews were transcribed verbatim. A health psychologist carried out the transcription, coding and initial interpretation of the data. A step-by-step analysis of the data followed, informed by interpretative phenomenological analysis (Smith and Osborn, 2003; Dean et al., 2006). All transcripts were read through twice to increase familiarity with the data before carrying out the analysis. During the third reading, statements of significance were coded (Smith and Osborn, 2003). This process was repeated for two further readings. In accordance with recommendations for increasing the rigour in qualitative medical research (Barbour, 2001), transcripts were also read and coded in the same manner by a consultant neurologist with a special interest in MND and a consultant health psychologist.

The three authors then discussed the emergent themes within the data. An iterative process of returning to explore the data and refine the list of themes was carried out. The three authors independently, and whilst blinded to themes of the other researchers, identified themes within the data. After a series of iterations, and following debate and discussion between the authors, a master list of themes emerged. Themes from the master list were then placed in groups belonging to superordinate domains that represented the patient experience of fatigue.

### Findings

#### Participant demographics

Ten patients (7 males, 3 females) were interviewed as part of this study. Mean age for the participants was 60 ± 14 years. Table 1 shows demographic and medical information for the interviewed population.

**Table 1:** **Demographic information for study participants**.

**Patient information**		**Percentage of symptom**	
		**presentation at time of interview**	
**Mean scale scores**		Bulbar	30%
ALSFRS-R	35.6 ± 7.3	Respiratory	40
HADS-D	3.7 ± 2.8	Arms	80
HADS-A	4.8 ± 3.3	Legs	80

Key: ALSFRS-R = Amyotrophic lateral sclerosis functional rating scale—revised (Cedarbaum et al., 1999); HADS = MND-validated Hospital Anxiety and Depression Scale (Gibbons et al., 2011a,b) D = Depression; A = Anxiety.

No patient was above the suggested threshold for “case-level” depression or anxiety, based on the modified cut-off points suggested for use in this population (Gibbons et al., 2011b) though one participant fell into the “doubtful” category for depression (Anne) and one for “doubtful” anxiety (Carole).

Analysis of the data revealed 15 themes, with three sub-themes that were then arranged into two superordinate domains of “Descriptions of fatigue” and “Effects of fatigue.” Themes are summarized in Table 2.

**Table 2:** **Domains, themes and subthemes within the dataset**.

**Domain**	**Theme**	**Sub-theme**
Descriptions of fatigue	Weakness	
	Tiredness	
	Energy	
	Concentration	
	Causal factors	
	Progression	
Effects of fatigue	Adaptation	Rest
		Reconceptualization
		Budgeting
		Planning
	Motivation	
	Avoidance	
	Frustration	
	Stress	

#### Opening statements

Answers to the first question in the interview schedule—*“Can you tell me what fatigue means to you, in your own words?”* generally described feelings of exhaustion, muscular weakness or having no energy. Bill describes the pervasive and disabling nature of his fatigue in his opening statement.

Just the tiredness, all the time tiredness, just no stamina. [] I haven't lost my strength but I have lost the ability to sustain it, I can't do simple things.

Other participants drew a clear distinction between fatigue experienced as reversible muscle weakness and fatigue experienced as whole-body tiredness.

[Mark] It is only in my arms and legs really, and it's there that I feel tired, not anywhere else really not in my mind or anything. I don't really feel weary; it's just arms and leg and muscle tiredness.

For many people, particularly those who experienced fatigue as predominately tiredness rather than reversible muscular weakness; the feelings of fatigue were extreme. Carole emphatically described her experience of fatigue:

Not *just* tired but *really* fagged out, you know. Got no energy whatsoever [] all I want to do is just lie down and rest.

#### Onset of fatigue

A common theme throughout the transcripts was that fatigue could be caused by only minimal exertion. Activities including handling cutlery, talking with friends on the telephone, going to the toilet and stepping up the front doorstep could elicit muscular weakness.

[Tom] Getting dressed now is an effort, if I get dressed and put my trousers on I have to stop now and catch my breath although I don't have breathing difficulties.

In the same way in which interviewees applied the word “*fatigue*” to both feelings of whole-body tiredness and muscular weakness, Mark and Frank used the word “*tiredness*” to describes the muscular fatigue they feel in their hands and limbs.

#### Energy

The experience of fatigue was commonly explained as lack of energy, analogies of being “*drained*” of energy or “*sapped*” were frequently offered. The concept of energy was applicable to both feelings of whole-body tiredness that Carole predominantly experienced, and the reversible muscular-weakness that was central to Mark's understanding of his fatigue.

[Carole] I don't do [anything] now. I do help my husband with the cooking sometimes and that usually saps the energy out of my and I'm usually only sitting on a stool saying “do that, do that.”

#### Rest

Accounts of fatigue were accompanied by a narrative about rest, or how the patient relieved his or herself from feeling fatigued.

For patients who experienced fatigue primarily as muscular weakness, rest was generally described as taking a few minutes to allow their muscles to recover, in line with the analogies of being “*drained*” of energy, rest was described as “*recharging*” the patient's energy levels.

[Mark] [I'd rest for] maybe three to four minutes, something like that. I make sure that it is long enough if it's any shorter then I'm back to square one; it feels like I am not recharged enough.

In contrast to the short, but frequent, rest periods adopted by patients experiencing muscular fatigue, feelings of tiredness were relieved much more slowly, with periods of rest lasting hours before recovery:

[Anne] if I get myself dressed in the morning then it will take a couple of hours to recover from getting dressed. [After that] I'd feel much better.

#### Concentration

Different accounts were given of fatigue-related difficulties in concentrating. The prominence of cognitive fatigue in other neurological disorders led to a specific exploration of the phenomenon in the interview schedule.

In some accounts, difficulty concentrating was linked to a difficulty in sustaining the physical effort component of the task the interviewee was struggling to concentrate on. Frank explained how he found it difficult to concentrate on magazines because he found them *“very heavy”* and resultantly felt fatigued whilst trying to read them.

Carole explains how her concentration is fine when she's watching television but since the onset of MND she has not read any books, finding them too difficult to concentrate on.

[Carole] [I find it difficult to concentrate] reading the paper or anything like that, in fact to be quite honest with you; since I got motor neurone disease or too ill I've never read a book and for someone who can read books one after the other. They are all there, I just can't read them. I just can't concentrate on them, television yeah, [television is fine].

[Interviewer] If you were to sit down and watch a programme lasting maybe an hour you'd feel fine watching that?

[Carole] Yes, that's no problem.

#### Causal factors—dyspnoea and talking

For two interviewees who suffered from breathing problem, fatigue had developed alongside difficulties in talking and breathing, just as it accompanied physical actions using their limbs. Frank describes that alongside other physical activity that tires him out, using bulbar musculature to talk has a similar effect on his fatigue.

Carole attributes fatigue that would otherwise be unexplained to the increased effort of breathing:

[Interviewer] Do you ever feel as though you are tired but can't really explain why?

[Carole] Yes I do sometimes, I must admit, but I put that down to that it being such an effort for me to breathe that I'm actually tiring myself out by breathing. When I am speaking I am using my lungs and that's where the effort is coming from.

Interestingly, in both these cases where fatigue was linked to difficulties with respiratory and bulbar involvement, it was relieved to some extent by the use of ventilation equipment. Frank explained that he only used his non-invasive ventilator when he felt fatigued. Frank's wife interjected to explain the effect the ventilator has after a busy weekend when the couple had spent time seeing their family.

[Frank's wife] and [he] put the mask on for two hours and when he's had his mask on it recharges him and he sat up last night for two and a half hours and that mask is really keeping him going and it's taking the tiredness away and sorting your oxygen levels out.

Frank emphatically confirmed the “boosting” effect of ventilation that his wife had described when prompted later in the interview.

#### Progression

A key emergent theme in the transcripts was progression, participants unanimously explained fatigue following a progressive course.

[Frank] It's getting worse, because prior to losing my legs I could do jobs and I may get tired in the evening but you'd probably be on the go all day doing various things.

Frank described his legs as being “lost” now in the sense that they had become functionally useless. This comment may seem severe but was presented as a mundane fact, indicating a stoic acceptance of this impairment.

John's contextualized the severity of the fatigue he felt by comparing it to his life prior to MND, explaining how the smallest of actions now are comparable to bouts of strenuous exercise prior to having MND.

[John] I used to play squash regularly and if you get into a rally and you know how tired you feel, it's really quite severe exercise and now you get that sort of feeling just by trying to stand up sometime and transfer from one chair to another, it's just … you can't believe it.

## Effects of fatigue

### Adaptation

#### Reconceptualization

Linked to the concept of progression was a narrative of adaptation to the illness, and the new demands that were placed on the patient as a result of their increased fatigue.

Reconceptualizing what constitutes physical exertion was a common feature of adapting to fatigue; Phil explains how he can't do anything that he had once considered to be a “normal thing”:

[Phil] I can't do anything that I would have considered “normal” before. It's just a matter of resting in the way of sitting in an armchair most of the day and only the odd movement from one room to another and that's about it.

John explains that things he used to take for granted now have a significant effect in terms of causing fatigue.

Well eating [makes me fatigued], and going to the toilet. [] generally just trying to stretch and pick up a cup or like pick up your food off a plate [] it's surprising now things that I never used to regard as physical are now becoming physical and causing fatigue.

#### Budgeting

Participants made reference to having to make adaptations in their daily lives in order to better budget daily levels of energy.

[Tim] [] you can only do so much with your arms each day sort of after that you either knacker yourself up for the next day or you become sort of rolling over in bed so you have to sort of time things.

The concept of budgeting energy throughout the day or planning prophylactic rest before a busy day was evident as an adaptive strategy employed by a number of patients.

[Tim] Once you have reached your limit for the day there are no more physical things to be done, then it becomes watching the television or reading… but no more physical effort.

#### Planning

Adaptation to fatigue was, for some, the adoption of prophylactic rest prior to, and acknowledging the need to rest after activities that the patient thought may cause fatigue, such as attending a clinic appointment or seeing going to visit relatives

[Joan] If I had a busy day coming up I'd make sure I wasn't doing anything before or after.

#### Motivation

This change in levels of available energy impacted participants, particularly in terms of reduced motivation to carry out activities of daily living (ADLs) that were expected to cause fatigue.

[Interviewer] Do you feel like you avoid things because they make you fatigued?

[John] Oh yes definitely, yeah. Definitely

[Interviewer] What sort of things?

[John] … sometimes it is just things like bothering to pick up the cup and have a drink you just think “I am comfortable sitting here where I am” and it seems stupid probably to you but at times you just think to yourself “if I do that I'm going to feel really *really* tired” and it seems stupid to me now, but I know it happens, it's amazing.

John's double reference to *“stupidity”* suggests that he feels some embarrassment in avoiding *“just simple things”* because of his anticipated fatigue. In a similar way Mark explains his adaptation to having to perform what was once a simple action in a number of steps, having adapted to incorporate a series of rests into his day-to-day life.

[Mark] Well first and foremost if there is something that I can't do anymore then I wouldn't attempt it. Err if it was something like taking my coat off I can't reach up and take my coat off straight away … I'd have to wait two or three minutes and *then* I'd be able to put the coat up, and then after I'd put the coat up I'd have to sit down. When you say activity I'm thinking hang a coat up on a coat hanger.

Tim describes a series of adaptations he is forced to undertake as the disease progresses. Tim's need to adapt was recognized and accepted with a matter-of-fact stoic attitude; as though it was just another job that needed to be done.

[Tim] It started in my left foot and has progressed down one leg and the other leg so that's put me in a wheelchair eventually and I now get round the house on this little three-wheeled scooter, which is also becoming a slight problem because now it's creeping up my body and arms are getting weaker and I'm using this chair to sort of get up, but it's adaptation and I need to keep adapting.

Fatigue affected respondents' motivation in different ways, in some domains motivation was markedly lower, whilst in others it was much higher. What was apparent in the transcripts was not necessarily a change in levels of overall motivation but a reprioritization, anchored in some cases to the patient's perceptions of a daily energy limit.

John describes how his motivation to use his PC has lessened because of the increased physical demands of operating the mouse and keyboard:

[John] I used to do quite a bit on the computer, I used to but well now I just find that because it's trying to key in and use the mouse you keep control of the mouse that is another one of those things that makes you tired, so I use the computer less and less which I do miss but it's something I can do without for a while. I just use it occasionally whereas before I was on it every day and quite often 7 hours a day but now I can leave it for a week and not touch it, it's that extent.

Feelings of fatigue and increasing physical impairment clearly made social contact require more effort, and the theme of social contact was closely mediated by motivation. John explains that despite a reduced motivation to use the computer, which he admits to missing, he makes a conscious effort to remain engaged in face-to-face social contact with his friends, even when he anticipates that social contact will lead to the same unpleasant feelings of fatigue that put him off doing other activities.

[John] My friends come to visit and we chat I think I manage to keep in the conversations and things like that and it's perhaps afterwards that I feel the effects then but during the period of doing it I am OK I suppose, it's got to be how much interest you have in that particular topic and the same with the computer it's easier to say it's not absolutely urgent but when you're sitting with friends and you're chatting you like to feel that you're part of that company so you make an effort to keep with them.

This desire to socialize is, as in John's narrative, set against descriptions of reduced motivation in other aspects of her life due to fatigue.

[Anne] it's more to do with motivation, it's a motivational tiredness rather than a physical tiredness; a lethargy, I can't be bothered more than anything.

Carole explained that she was still motivated to carry on with activities, even if they prompted feelings of being “shattered.” Her increased motivation appears to stem from a desire to “keep her life” and maintain her independence by dressing without the assistance of her husband.

[Carole] The motivation is there, the brain is willing; it's my body that is weak. That's why I get ratty with myself and with other people because I can't do what I want to do. I do try, but it just doesn't work. I try to force myself to do things but it doesn't work. Well if [my husband] is in the bathroom and I'm in the bedroom getting dressed. Which I have to do and then he comes in and gets annoyed and says “*Why didn't you wait those few minutes?*” He says…”*Look, you're shattered now*”

[Interviewer] Do you feel it is important to keep on trying?

[Carole] Well I have to keep a life, don't I?

This willingness to keep on “*fighting*” against the increased fatigue was apparent elsewhere. When questioned if he avoided activities that he expected would make him fatigued, Tom boldly replied: *“No no, I have to fight it.”*

#### Avoidance

Patients throughout the sample explained how subtle changes in their motivation had occurred due to living with the illness and their experiences of fatigue. John explains that he developed a learned response to avoid activities that he knew would make him feel the effects of fatigue.

[John] [The fatigue feels] quite debilitating really, in certain areas and at certain times anyway it makes you feel as though you can't do anything for quite a time and you just have to sit and relax and try to recompose yourself for ten or fifteen minutes.

[Interviewer] How will you feel after that?

[John] Better, but the thing is… because of what's happened to you to get that fatigue you are less likely to want to do those sorts of things anyway.

#### Frustration

Frustration was commonly experienced due to the increased difficulty, or impossibility, in doing ADLs conflicting with continued desire to stay engaged in ADLs.

[Anne] It's more frustrating than anything else, not being able to do what I want to do, especially with the kids. Or frustrated that you can't get yourself dressed or it takes you half an hour to get up and what have you.

#### Stress

The emotional burden of stress was described as being an aggravating factor for fatigue and was described in a number of interviews.

[John] I am certainly more tired [when I am stressed] but not to the extent as when [I'm] doing something physical.

## Discussion

The data revealed that the experience of fatigue in MND differs between individuals and appeared to be multi-factorial, including perceptions of muscular fatigue, whole-body tiredness and to a lesser extent, difficulties concentrating. There was no evidence that patients believed fatigue to be caused by anything other than the natural progression of the disease itself (i.e., not from difficulty sleeping).

The primary experiences of fatigue were constant exhaustion and use-dependent muscular weakness. Use-dependent reversible muscle weakness appears an important issue in MND, highlighting the need to move beyond Ream and Richardson's (1996) definition of fatigue for nursing.

The data revealed feelings of constant exhaustion that could occur separately from use-dependent reversible muscular weakness. This whole-body tiredness was overwhelming and forced the interviewees to engage in long periods of rest.

Participants that experienced these two forms of fatigue often used the same language of feeling “fatigued” or experiencing “tiredness” to describe very different feelings. This highlights the importance of considering context in the interchangeable descriptions of patient fatigue as these two different forms of fatigue are often described using the same terminology. It is crucial therefore that clinicians and researchers understand that interchangeable terminology may be used when describing fatigue, although the two concepts of reversible muscular weakness and whole body tiredness are considered to be distinct by patients.

Bulbar weakness and breathing difficulties appeared to have an effect of some aspects of fatigue and may be closely related developmentally. Of particular interest were accounts of fatigue being relieved by use of non-invasive ventilation, as has been explored elsewhere (Bourke et al., 2003), and fatigue worsening when symptoms of breathlessness were more prevalent, as mentioned earlier, in hot weather. The relationship between fatigue and dyspnoea, two important features of this disease, has not been fully explored and warrants further examination.

The severity of fatigue and progressive reduction in energy expenditure that could occur before feelings of fatigue were present was well described in the data. These data confirm the importance of fatigue in MND (Chio et al., 2004) as well as its persistent progressive nature. Regardless of how fatigue presented itself in the patients, all patients adopted adaptive strategies.

Alongside narrative accounts of what fatigue *is*, patients described the effects of fatigue on their lives. Adaptation was a key prevalent theme, with three subthemes of reconceptualizing, budgeting and planning. The relentless progression of fatigue forced patients to make adaptive changes in their lifestyle. Patients explained how they found it difficult doing “the simple things” and began to understand actions such as hanging up a coat as requiring a significant amount of effort.

As well as reconceptualizing the difficulty tasks, patients adapted by adopting strategies of budgeting daily energy expenditure and planning around fatigue. Prophylactic rest was a commonly employed by patients who were expecting to expend more energy than they usually would, such as before a clinic visit. Budgeting energy was an adaptation strategy of that was used on a daily basis. Because of the inevitable fatigue that would occur in the afternoons, many patients tried to do all of their daily activities in the morning, and were cautious not to “overspend” their daily budget.

The amount of effort that was required to accomplish such tasks impacted on patient motivation in a number of ways. Patients sometimes avoided actions, like picking up a cup of coffee because a sip of a drink could not justify the inevitable fatigue that would follow.

Interestingly, descriptions of reduced motivation to do task that would lead to fatigue were followed by explanations of how patients felt more motivated to integrate socially with friends and §s in particular reported that worry and stress contributed to fatigue. Feelings of frustration were often described when patients were unable to do what they wanted to do, because doing so would leave them feeling fatigued.

### Limitations of the study

There are a number of limitations inherit in all qualitative research. The current study is limited as the results may not be fully generalized to other patient populations where fatigue is an issue, such as patients with myasthenia gravis. Similarly, the relatively small sample sizes required for IPA research may mean that the lived experiences and descriptions of fatigue illustrated in the analysis may not be reflected in other groups with MND, although the degree of internal consistency within the interviewed sample that suggested they may be comprehensive.

### Definition of fatigue

From these findings, fatigue in MND can be defined as “Reversible motor weakness and whole-body tiredness that was predominantly brought on by muscular exertion and was partially relieved by rest.”

## Conclusion

Fatigue appears to be primarily experienced in two ways by patients with MND, and could be experience as use-dependent reversible muscle weakness or feelings of whole-body tiredness, or a combination of the two. It is essential that clinicians understand this dichotomy in MND fatigue and appreciate the semantic issues involved with fatigue, especially that patients may interchangeably use words such as “fatigue” and “tiredness” to describe essentially different experiences of fatigue.

### Conflict of interest statement

The authors declare that the research was conducted in the absence of any commercial or financial relationships that could be construed as a potential conflict of interest.

## Appendix 1: interview question schedule

Can you tell me what you understand by the term fatigue?

   What does fatigue mean to you?

   <Compare to muscular weakness?>

   What does fatigue mean to you?

   How does it change from day to day?

   How does fatigue feel?

   How do you recover from fatigue? Do you rest?

   How do you feel after a meal?

   How does you coordination change when you become fatigued?

   When you are tired, do tasks seem harder to complete?

   Does fatigue affect you concentration?

   Would you become fatigued from little physical exertion, for example sitting and watching TV or reading a book (do you need to be “exercising” to become fatigued)

   Ok considering all this, can you describe to me what you do on a “typical” day?

   Do you often feel the need to rest?

   How long would you rest for?

   Would that totally relieve the fatigue?

   For how long?

   For how long are you sleeping at night?

   Is you sleep undisturbed?

   Do you wake in the night for no reason?

   And when you wake up how do you feel?

   In what parts of your body do you mainly feel fatigued?

   What things do you do that make you fatigued?

   What things don't you do, because you think they may make you fatigued?

   Do you avoid going out when fatigue is bad?

   Do you feel fatigued if you exert yourself mentally, or concentrate on something for a long time?

   Does the weather have any effect on how you feel?

   Does the hot or the cold affect you?

   How is your concentration when it is hot?

   Are there things you can't do anymore because of fatigue

   How is your speech when you are tired?

   Do you yawn a lot more than before?

   If you were going to do something that required a lot of physical effort, how would you prepare?

   What were the symptoms that you realized were occurring that eventually led to your diagnosis?

   How did these symptoms develop leading up to diagnosis?

   How did these symptoms change after you started to receive treatment?
